# Near-Infrared Reflectance Spectroscopy for Quantitative Analysis of Fat and Fatty Acid Content in Living *Tenebrio molitor* Larvae to Detect the Influence of Substrate on Larval Composition

**DOI:** 10.3390/insects14020114

**Published:** 2023-01-23

**Authors:** Nina Kröncke, Monique Neumeister, Rainer Benning

**Affiliations:** Institute of Food Technology and Bioprocess Engineering, University of Applied Sciences Bremerhaven, 27568 Bremerhaven, Germany

**Keywords:** near-infrared reflectance spectroscopy, *Tenebrio molitor*, nutritional composition, fatty acids, edible insects, lipids

## Abstract

**Simple Summary:**

Insects are an important source of protein but insect fat increasingly slides into focus due to its high content of unsaturated fatty acids. *Tenebrio molitor* larvae were reared on several substrates with different nutritional contents to influence the fat and fatty acid contents of the larvae. The fat and fatty acid composition of the mealworm larvae were then analyzed in order to determine if a nutritional change could be detected using near-infrared reflectance spectroscopy. This is a rapid and non-destructive method for the online analysis of chemical composition. In this study, the diet used for rearing had a significant effect on the larval fat content and fatty acid composition as well as the absorbance of the near-infrared spectra and the larval growth rate and weight gain, i.e., a high fat content substrate reduced weight gain and larval growth. The most prevalent fatty acids identified and quantified were palmitic, oleic, and linoleic acid, showing a correlation between larval content and rearing diets containing high fatty acid amounts. A high dietary content of lauric acid, myristic acid, and α-linolenic acid resulted in a high content of these fatty acids in mealworm larvae. The fat and fatty acid content could be predicted accurately using near-infrared reflectance spectroscopy and were highly influenced by several diets having different proximate compositions.

**Abstract:**

Several studies have shown that mealworms (*Tenebrio molitor* L.) could provide animals and humans with valuable nutrients. *Tenebrio molitor* larvae were studied to determine whether their rearing diets affected their fat and fatty acid content and to ascertain if it is possible to detect the changes in the larval fat composition using near-infrared reflectance spectroscopy (NIRS). For this reason, a standard control diet (100% wheat bran) and an experimental diet, consisting of wheat bran and the supplementation of a different substrate (coconut flour, flaxseed flour, pea protein flour, rose hip hulls, grape pomace, or hemp protein flour) were used. The results showed lesser weight gain and slower growth rates for larvae raised on diets with a high fat content. A total of eight fatty acids were identified and quantified, where palmitic, oleic, and linoleic acids were the most prevalent and showed a correlation between larval content and their content in the rearing diets. There was a high content of lauric acid (3.2–4.6%), myristic acid (11.4–12.9%), and α-linolenic acid 8.4–13.0%) in mealworm larvae as a result of the high dietary content of these fatty acids. NIR spectra were also influenced by the fat and fatty acid composition, as larval absorbance values differed greatly. The coefficient of the determination of prediction (R^2^_P_) was over 0.97, with an RPD value of 8.3 for the fat content, which indicates the high predictive accuracy of the NIR model. Furthermore, it was possible to develop calibration models with great predictive efficiency (R^2^_P_ = 0.81–0.95, RPD = 2.6–5.6) for all fatty acids, except palmitoleic and stearic acids which had a low predictive power (R^2^_P_ < 0.5, RPD < 2.0). The detection of fat and fatty acids using NIRS can help insect producers to quickly and easily analyze the nutritional composition of mealworm larvae during the rearing process.

## 1. Introduction

The world population has been increasing for decades and is forecast to reach 9.7 billion people by 2050 [1]. Therefore, food production must be increased by up to 70% to ensure the supply of food for the world’s population [2]. Insects have been used as a food source by humans for a long time and are consumed by about two million people worldwide [3]. In 2021, dried *Tenebrio molitor* larvae were approved as a novel food by the European Food Safety Authority (EFSA) in the EU. Insects are a good alternative and sustainable food source due to their nutritional composition and environmental advantages [2]. Livestock farming is a major contributor to global climate change [4], whereas insects produce lower greenhouse gas emissions, especially compared to cattle and pigs [5]. Furthermore, they have a lower water footprint and land requirements, as well as a good feed conversion rate [4,5]. Insects have a very high edible content of 80–100%, being much higher than other livestock (40–50%) [5]. In addition, they have a high protein (25–75%) and fat (10–70%) content in dry matter and are rich in essential amino acids and polyunsaturated fatty acids [6,7,8]. The composition and growth of mealworm larvae depend on rearing conditions, e.g., environmental influences such as temperature and relative humidity [9]. Their diet, too, has a significant influence, whereby the nutrient composition of mealworm larvae can be regulated by targeted feeding. For example, supplementing the diet with flaxseed meal and oil increased the content of α-linolenic acid in mealworm larvae [8,10]. Therefore, research on nutritional requirements, transport of nutrients, and metabolism, especially dietary lipid utilization and storage, has become increasingly important [9]. Consequently, and for food safety and quality control reasons, it is beneficial to monitor nutritional parameters such as fat and fatty acid content during rearing. Standard methods of analysis are usually time-consuming, expensive, and environmentally harmful because of the use of chemicals [11]. Near-infrared reflectance spectroscopy (NIRS) is a fast method for the quantitative determination of the moisture [12], macronutrient (protein and fat) [13,14], and micronutrient (amino and fatty acids) [15,16] content for simultaneous analysis without extensive sample preparation [11]. Further advantages are the easy handling, non-destruction of the sample, and the possible integration of the analysis into the rearing process [11]. NIRS has been applied in the food industry for process and quality assurance for a long time; studies have been published on the use of NIRS for the identification of insects in cereal grains [17] or on the detection of insect fragments in wheat flour [18]. In addition, the fat or fatty acid content of meat [19], cereals and their products [20], or cocoa beans [21] can already be predicted by NIRS. As we have demonstrated in our previous study [22], NIRS can be used to predict the moisture and protein content of mealworm larvae. Thus, the present work focused on determining the fat content and fatty acid composition of living *Tenebrio molitor* larvae using NIRS due to the deficiency of known applications for such measurements. The aim was to develop multivariate classification models based on the reference data and NIR spectra to allow for an accurate prediction of the fat and fatty acid content in living *Tenebrio molitor* larvae. Furthermore, the influence of diet on the fat and fatty acid content of mealworm larvae was analyzed. Since the fat synthesis of mealworm larvae can occur from dietary lipids, proteins, and carbohydrates, their contents were varied in the diets [23,24]. 

## 2. Materials and Methods

### 2.1. Insect Samples

*Tenebrio molitor* larvae were reared at the University of Applied Sciences Bremerhaven at 27 °C and a relative humidity of 75% in a constant climate chamber (HPP 110, Memmert, Schwabach, Germany) and were fed with wheat bran ad libitum until the larvae reached an age of eight weeks. All experimental groups consisted of 100 larvae with an average start weight of 6.1 ± 0.6 mg per larva. These were placed in 400 mL beakers and weighed (ADB 200-4, Kern & Sohn GmbH, Balingen-Frommern, Germany) at the beginning and the end of the experiment so that the biomass increase could be recorded. The period of the feeding trial was five weeks. After collecting the final weights, the mealworm larvae were placed in empty beakers, starved for 24 h, and measured using NIRS. The frass and remaining feed were collected and weighed to calculate food utilization. Dead larvae were counted to determine the survival rate. Larvae were euthanized at the end of the experiment by freezing at −21 °C for 48 h using a freezer (HAS 47520, Beko, Neu-Isenburg, Germany) and stored until the fat and fatty acid analyses were carried out.

### 2.2. Feeding Treatments

A variety of substrates were selected due to their differences in nutritional content as indicated according to the manufacturer (see Supplementary Material Table S1). Groups were combined based on the macronutrient content, in particular, the carbohydrates, proteins, and fats, of the substrates which are shown in Table 1. The treatments needed to contain a substrate with a high content of fats, proteins, and carbohydrates, so that the influence on growth and nutritional composition, and especially on the fat content, of *T. molitor* larvae could be examined. In addition, the influence on the fatty acid composition of the larvae should be investigated. Therefore, substrates were also chosen because of their differences in fatty acid profile. The following ingredients were selected for the experimental diets, based on their nutritional composition and included: coconut flour (Fischmix, Iserlohn, Germany), flaxseed flour (Fischmix, Iserlohn, Germany), rose hip hulls (Holger Senger Vertrieb von Naturrohstoffen e.K., Dransfeld, Germany), grape pomace (Holger Senger Vertrieb von Naturrohstoffen e.K., Dransfeld, Germany), hemp protein flour (Demeterhof Schwab GmbH & Co. KG, Windsbach, Germany), pea protein flour (Raab Vitalfood GmbH, Rohrbach, Germany), and wheat bran (Roland Mills United GmbH & Co. KG, Bremen, Germany). The substrates were mixed with wheat bran to create groups with different nutritional contents (Table 2). However, the fat content was primarily the focus of this study and therefore the groups were named according to the substrate with fat content (e.g., the CF5 group consists of coconut flour and wheat bran with a total fat content of 5%). Further groups are: coconut flour and wheat bran with 10% fat (CF10), coconut flour and wheat bran with 15% fat (CF15), coconut flour and wheat bran with 20% fat (CF20), flaxseed flour and wheat bran with 5% fat (FSF5), flaxseed flour and wheat bran with 10% fat (FSF10), flaxseed flour and wheat bran with 15% fat (FSF15), flaxseed flour and wheat bran with 20% fat (FSF20), grape pomace and wheat bran with 4% fat (GP4), hemp protein flour and wheat bran with 5% fat (HPF5), hemp protein flour and wheat bran with 8% fat (HPF8), rose hip hulls and wheat bran with 4% fat (RHH4), pea protein flour and wheat bran with 5% fat (PPF5), and pea protein flour and wheat bran with 6% fat (PPF6). The control group consisted of pure wheat bran (WB control). Each beaker contained 10 g of the diet and 3 g of carrot as a water source, which was given once a week, with five replicated beakers per feeding group.

### 2.3. Calculations

The biomass increase, measured as the larval weight gain per larvae (LWGpL), specific growth rate (SGR), feed conversion ratio (FCR), and conversion efficiency of ingested food (ECI) were determined after the feeding experiment and calculated based on the following formulas [25]:

(1) $$\mathrm{LWGpL}=\frac{\mathrm{Larval}\;\mathrm{weight}\;\mathrm{end}\;-\;\mathrm{Larval}\;\mathrm{weight}\;\mathrm{start}\;}{\mathrm{Number}\;\mathrm{of}\;\mathrm{larvae}\;\mathrm{at}\;\mathrm{beginning}\;-\;\mathrm{Number}\;\mathrm{of}\;\mathrm{dead}\;\mathrm{larvae}}$$

(2) $$\mathrm{FCR}=\frac{\mathrm{Feed}\;\mathrm{consumed}}{\mathrm{Weight}\;\mathrm{gained}}$$

(3) $$\mathrm{ECI}=\frac{\mathrm{Weight}\;\mathrm{gained}}{\mathrm{Feed}\;\mathrm{consumed}}\;\times \;100\%$$

(4) $$\mathrm{SGR}=\frac{\ln\;\mathrm{Final}\;\mathrm{body}\;\mathrm{weight}-\;\ln\;\mathrm{Initial}\;\mathrm{body}\;\mathrm{weight}}{\mathrm{Experimental}\;\mathrm{days}}\;\times \;100\%$$

### 2.4. Analysis of Fat Content

The fat content of mealworm larvae is represented as g/100 g of fresh weight and was analyzed using the Soxhlet method as described by the Association of German Agricultural Investigation and Research Institutions [26] with petroleum benzine (Carl Roth GmbH & Co. KG, Karlsruhe, Germany) as the extraction solvent. The extracted fat samples were used for the fatty acid analysis and stored at −21 °C until use. 

### 2.5. Methylation and Analysis of Fatty Acid Composition

Methylation of fatty acids (FA) was performed according to DIN EN ISO 12966-3 and described by the Association of German Agricultural Investigation and Research Institutions [26]. The extracted fat sample (10 mg) was weighed into a screw-top glass test tube and methyl-tert-butyl ether (500 µL, MTBE, Merck KGaA, Darmstadt, Germany) and trimethylsulfonium hydroxide in methanol (250µL, TMSH, Carl Roth GmbH & Co. KG, Darmstadt, Germany) were added and vortexed at high speed for 30 s. The FA methyl esters (FAMEs) were then transferred into a sample vial (2 mL) and injected directly into the gas chromatograph (GC). A GC endued with a flame ionization detector (Trace 1300, Thermo Fisher Scientific, Waltham, MA, USA) was used to analyze the fatty acid content of the pure substrates (see Supplementary Material Table S2) and mealworm larvae (Table 3). The column oven temperature program was set to 130 °C at the start, heated 2 °C/min to 230 °C, and injected at 240 °C with a 10:1 inlet split ratio. The ionization detector port was set at 250 °C. Helium was used as a carrier gas and its flow was maintained at a constant 1.1 mL/min throughout. The identification of peaks was established by comparing retention times with known FAME standards (Supelco 37 component fame mix, Merck KGaA, Darmstadt, Germany). The percentage composition of the fatty acid compound in the mixture was received from the percentage peak area obtained using the GC data system software (Chromeleon 7.2, Thermo Fisher Scientific, Waltham, MA, USA). The individual fatty acids of mealworm larvae and single substrates detected from fatty acid composition analysis were reported as a percentage of the total fatty acids, and the results were presented as the mean ± standard deviation of the duplicate experiment (*n* = 2).

### 2.6. Statistical Analysis

Statistical analyses were performed in SigmaPlot 12.5 (Systat Software Inc., Düsseldorf, Germany). Growth and feed utilization parameters were checked for normality and homogeneity of variances. Significant differences (*p* < 0.05) in larval weight gain, growth rate, feed conversion, and efficiency were assessed using the one-way ANOVA and Tukey–Kramer post hoc test. The fat and fatty acid content of the larvae was determined by duplicate, so no statistical evaluation was performed.

### 2.7. Near-Infrared Spectra Collection

A near-infrared reflectance spectrometer (PSS 2120, Polytec GmbH, Waldbronn, Germany) was used to analyze *Tenebrio molitor* larvae. The NIR spectra were recorded at a wavelength from 1100 to 2100 nm. All samples were measured in quintuplicate (*n* = 5) and included the NIR spectra averaged from 50 spectra in total per sample.

### 2.8. Chemometrics

Matlab (version R2020a, The MathWorks Inc., Natick, MA, USA) and the PLS Toolbox (version 8.9.1, Eigenvector Research Inc., Wenatchee, WA, USA) were used to create NIR prediction models for the fat and fatty acid content of living mealworm larvae. Two sets of samples (calibration and validation) were required to develop a calibration model for a NIRS application for the quantification of the fat and fatty acid content of mealworm larvae. The calibration set included all samples proposed for inclusion in the data library and was used for creating the calibration model. The validation set should be completely independent of those samples applied to create the data library and is used to verify the predictive accuracy of the calibration model. Consequently, samples were divided into two subsets, a calibration set (*n* = 80) and a validation set (*n* = 40). The prediction models were developed using partial least square (PLS) regression in order to describe the differences in the fat and fatty acid content of mealworm larvae. Mathematical pretreatment methods, such as multiple scatter correction (MSC), detrend, mean centering (MC), and first (1D) and second (2D) derivatives, were used for processing the raw NIR spectra. The maximum value of variance was used to define the optimal number of latent variables (LV). The prediction accuracy and practical utility applicability of the prediction model were evaluated based on the coefficient of determination for calibration (R^2^_C_), root mean square error of calibration (RMSEC), coefficient of determination in prediction (R^2^_P_), root mean square error of prediction (RMSEP), coefficient of determination describing the correlation of relative FA concentration with the total fat content (R^2^_F_), and the ratio of performance to deviation (RPD).

## 3. Results

### 3.1. Larval Growth and Feed Conversion Parameters

**Figure 1:**
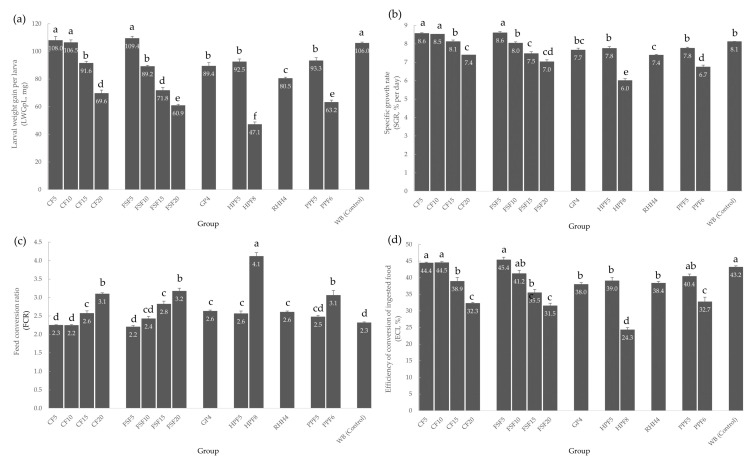
(**a**) Larval weight gain per larva, (**b**) specific growth rate, (**c**) feed conversion ratio, and (**d**) efficiency of conversion of ingested food of *Tenebrio molitor* larvae from different feeding groups; CF5: coconut flour and wheat bran (5% fat); CF10: coconut flour and wheat bran (10% fat); CF15: coconut flour and wheat bran (15% fat); CF20: coconut flour and wheat bran (20% fat); FSF5: flaxseed flour and wheat bran (5% fat); FSF10: flaxseed flour and wheat bran (10% fat); FSF15: flaxseed flour and wheat bran (15% fat); FSF20: flaxseed flour and wheat bran (20% fat); GP4: grape pomace and wheat bran (5% fat); HPF5: hemp protein flour and wheat bran (5% fat); HPF8: hemp protein flour and wheat bran (8% fat); RHH4: rose hip hulls and wheat bran (4% fat); PPF5: pea protein flour and wheat bran (5% fat); PPF6: pea protein flour and wheat bran (6% fat); and WB: wheat bran (control). Differences between treatments (*p* < 0.05) are indicated by different letters, results are given as the mean ± standard deviation, *n* = 5.

The growth performance and food utilization of *Tenebrio molitor* larvae are illustrated in Figure 1. Mealworm larvae fed on a diet of flaxseed flour and wheat bran with a fat content of 5% (FSF5) showed the highest LWGpL (109.4 ± 1.4 mg) and SGR (8.6 ± 0.1%), followed by the diet mixture of coconut flour with 5% fat (CF5) at 108.0 ± 2.9 mg (LWGpL) and 8.6 ± 0.0% (SGR) and coconut flour with 10% fat (CF10) at 106.5 ± 1.9 mg (LWGpL) and 8.5 ± 0.0% (SGR), although no significant differences (*p* > 0.05) exist between the treatments. On the other hand, *Tenebrio molitor* larvae fed with hemp flour and wheat bran with a fat content of 8% (HPF8) showed the lowest LWGpL (47.1 ± 1.8 mg) and SGR (6.0 ± 0.1%) to a significant degree (*F* = 5.73, df = 14; *p* = 0.001). Likewise, a similar trend was noticed on the ECI. In terms of the food conversion ratio, HPF8 showed the highest FCR (4.1 ± 0.1) among all, while the groups CF5, CF10, and FSF5 had a very low FCR (2.2–2.3) and constituted the most efficient diets. The control group, consisting of pure wheat bran, was comparable in all parameters with the three best diets (CF5, CF10, and FSF5), with no significant differences, except for SGR (*F* = 1.35, df = 14; *p* = 0.001). The survival rate was over 99% in all groups.

### 3.2. Fat Content of Mealworm Larvae

**Figure 2:**
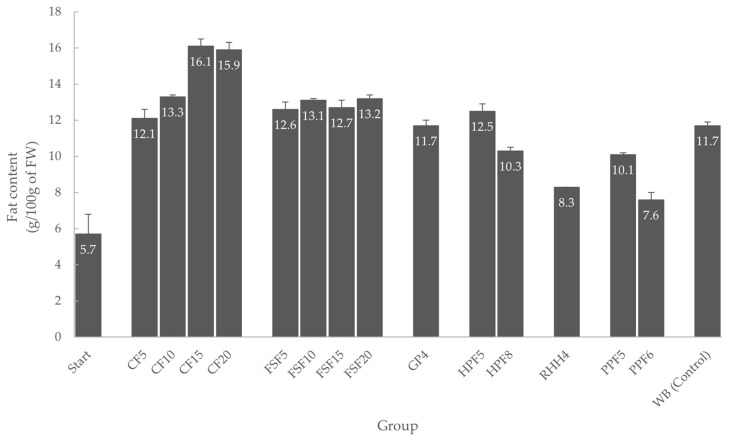
Fat content (g/100 g of fresh weight basis, mean ± standard deviation, *n* = 2) of *Tenebrio molitor* larvae from different feeding groups; Start: fat content of the larvae at the beginning of the experiment; CF5: coconut flour and wheat bran (5% fat); CF10: coconut flour and wheat bran (10% fat); CF15: coconut flour and wheat bran (15% fat); CF20: coconut flour and wheat bran (20% fat); FSF5: flaxseed flour and wheat bran (5% fat); FSF10: flaxseed flour and wheat bran (10% fat); FSF15: flaxseed flour and wheat bran (15% fat); FSF20: flaxseed flour and wheat bran (20% fat); GP4: grape pomace and wheat bran (5% fat); HPF5: hemp protein flour and wheat bran (5% fat); HPF8: hemp protein flour and wheat bran (8% fat); RHH4: rose hip hulls and wheat bran (4% fat); PPF5: pea protein flour and wheat bran (5% fat); PPF6: pea protein flour and wheat bran (6% fat); and WB: wheat bran (control).

The fat contents of *Tenebrio molitor* larvae are presented in Figure 2 and ranged between 5.7 and 16.1 g/100 g. The larvae at the beginning of the experiment had the lowest fat content (5.7 ± 1.1 g/100 g), which subsequently increased in all groups during feeding. The highest fat content (16.1 ± 0.4 g/100 g) was reached by the larvae fed with coconut flour and wheat bran (CF15), followed by the group CF20, where *T. molitor* larvae reached a fat content of 15.9 ± 0.4 g/100 g. Compared to the control group (WB), the groups with a higher substrate fat content (coconut flour and flaxseed flour) were able to obtain a higher fat content in the larvae. The lowest fat content (7.6 ± 0.4 g/100 g) was achieved by group PPF6, which was fed with pea protein flour and wheat bran.

### 3.3. Fatty Acid Composition of Mealworm Larvae

The results presented in Table 3 show a wide range of variability in the fatty acid content of *Tenebrio molitor* larvae and the influence of the fatty acid composition of the substrate. Detailed information on the fatty acid composition of the different feeding groups can be found in Supplementary Material Table S3. The enrichment with coconut flour, which has a high content of lauric acid (50.8 ± 0.2%) and myristic acid (18.3 ± 0.3%), resulted in very high increasing values of lauric acid and myristic acid in the groups CF10 (4.6 ± 0.1% C12:0; 12.2 ± 0.7% C14:0), CF15 (4.7 ± 0.1% C12:0; 12.9 ± 0.7% C14:0), and CF20 (3.2 ± 0.1% C12:0; 11.4 ± 0.4% C14:0), compared to all other groups (<1.0 ± 0.1% C12:0; <6.4 ± 0.6% C14:0). Coconut flour enrichment also resulted in high levels of palmitoleic acid (2.4 ± 0.1%) in CF10 and oleic acid (44.8 ± 0.1%) in CF20, and the supplementation of pea protein flour increased the stearic acid content (6.7 ± 0.2%) in PPF6 noticeably. The content of linoleic acid was highest (34.8 ± 6.3%) in the initial larvae and varied greatly depending on the feeding. Very low values (16.3 ± 0.6) of linoleic acid were reached by the group CF20 fed with coconut flour and wheat bran, whereas the control group WB, which was fed without supplementation, reached a higher content (30.3 ± 0.4%). The FA composition of the flaxseed flour used was reflected in the composition of essential fatty acids in the fat of mealworm larvae. Since flaxseed has a high content of α-linolenic acid (43.5 ± 0.3%; see Supplementary Material Table S2), very high levels of α-linolenic acid were also detected in the larvae fed with flaxseed flour, especially in the groups FSF10 (8.4 ± 0.7%), FSF15 (13.0 ± 0.3%), and FSF20 (10.5 ± 0.0%).

There was a wide variation in the percentages of the saturated fatty acid (SFA), monounsaturated fatty acid (MUFA), and polyunsaturated fatty acid (PUFA) content of the fat from *Tenebrio molitor* larvae fed on pure wheat bran and those fed with enriched substrates (Table 3). In the control group WB, the content of UFAs (39.1 ± 0.6% MUFA, 31.7 ± 0.5% PUFA) was higher than the SFAs (29.3 ± 1.1%). The most abundant SFA was found in palmitic acid (21.7 ± 0.1%), whereas oleic acid (37.4 ± 0.6%) and linolenic acid (30.3 ± 0.2%) were the most prevalent UFAs. When the feed was supplemented, it was observed that the SFA content in the groups with flaxseed flour was lower (26.3–28.2%) compared to the control group WB (29.3 ± 1.1%). Very high SFA values of over 40% could also be detected, especially in the groups CF10 (41.4 ± 0.9%), CF15 (40.4 ± 0.9%), and CF20 (40.0 ± 0.4%) fed with coconut flour. The PUFA and MUFA contents of *T. molitor* larvae also varied (Table 3). The highest PUFA value of 46.5 ± 0.1% was recorded in larvae fed with CF20, which also had the lowest MUFA value (16.6 ± 0.5%). In comparison with the control group WB, the diets enriched with coconut and flaxseed flour were able to achieve the highest PUFA and MUFA contents.

### 3.4. Near-Infrared Spectra

**Figure 3:**
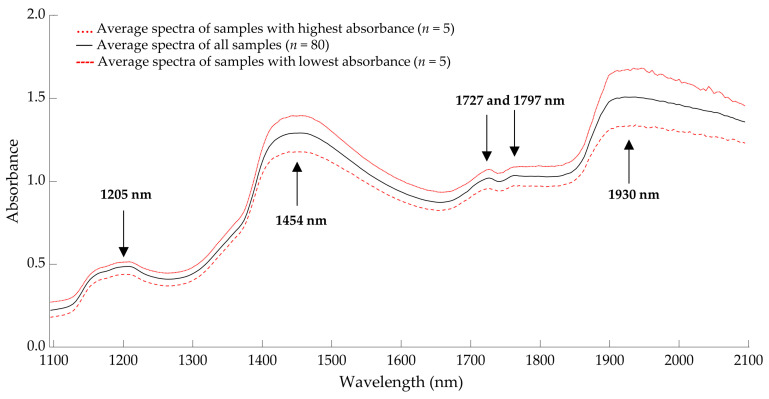
Average spectral data of living mealworm larvae from samples with the lowest (*n* = 5) and highest (*n* = 5) absorbance compared to all samples (*n* = 80).

The average spectral raw data of living *Tenebrio molitor* larvae are presented in Figure 3. Further details of the near-infrared raw spectra of all groups can be found in the Supplementary Material (Figure S1). Five different peaks can be identified at a wavelength of 1205, 1454, 1727, 1797, and 1930 nm. The near-infrared absorption bands around wavelengths 1205, 1727, and 1797 nm are related to fat content [19,27]. Differences in water content are usually visible at wavelengths of 1450 and 1950 nm [27,28], as well as fluctuations in protein molecules [27,29,30]. Hydrogen bonds have a higher absorption intensity in the near-infrared region and therefore dominate the NIR spectrum compared to other molecular bonds [31].

### 3.5. Prediction Model of Larval Fat Content

Table 4 shows the descriptive statistics for the fat content of living mealworm larvae in the calibration and validation sets. The data represent a range of variability in the fat content (7.4–16.2 g/100 g), which can be useful for a high prediction accuracy [11]. No outliers were detected during the calibration development.

Table 5 represents the PLS model performance of the calibration and validation set of the fat content in living mealworm larvae based on NIR raw spectral data using different mathematical pretreatments. The calibration model without preprocessing does not provide the best prediction. Furthermore, using the first and second derivatives was unable to improve the model and the application of multiple scatter correction improved the prediction only slightly. However, by applying mean centering, the predictability of the PLS model led to the best results with a high RPD value (8.33), indicating a robust calibration that is adequate for routine analysis [32,33].

**Figure 4:**
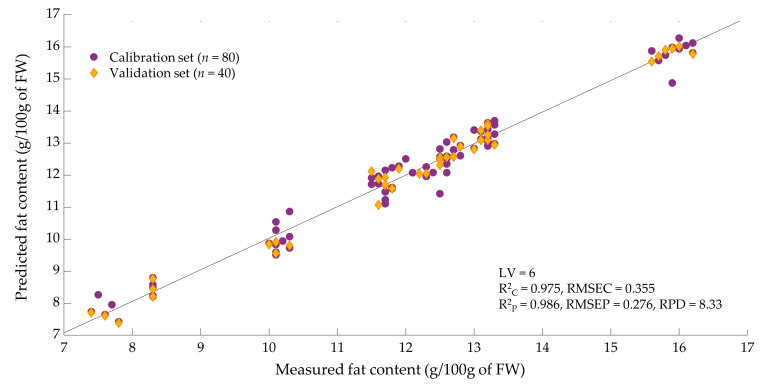
Comparison of the measured and predicted values of the fat content of living *Tenebrio molitor* larvae in the calibration and validation sets.

The best PLS prediction model for fat content was chosen in terms of a high R^2^_C_, R^2^_P_, and RPD, a low RMSEC and RMSEP, and the limiting number of factors (<10); this is presented in Figure 4. Table 5 shows the prediction models for the fat content of living mealworm larvae. Mathematical processing of the raw spectral data is necessary to increase the precision of the PLS prediction model. The highest prediction validity of the fat content was observed with the mean centering pretreatment (R^2^_P_ = 0.986, RMSEP = 0.276, RPD = 8.33).

### 3.6. Prediction Model of Larval Fatty Acid Content

Calibration and validation data for the fatty acid content of living *Tenebrio molitor* larvae are presented in Table 6. Fatty acids showed a wide range of variability, except for palmitoleic acid (1.1–2.5%). In addition, the content of SFA (25.8–42.0%), MUFA (28.6–46.6%), and PUFA (16.2–41.1%) varied strongly. The prediction quality was developed without detecting any outliers.

The NIR prediction models (Table 7) for the fatty acid content in mealworm larvae are based on the raw NIR spectra in calibration and validation sets, while differential mathematical treatments were used. Accordingly, the best model was selected, based on a high RPD and R^2^_P_ value, a low RMSEP, and a limited number of latent variables (<10). The multiple scatter correction (MSC), detrend, and second derivative (2D) were considered as suitable preprocessing methods for the fatty acid spectral data. The standard error of cross-validation of the PLS calibration was used to determine the optimal combination of algorithm parameters. The best results were obtained with MSC for myristic acid, oleic acid, linolenic acid, and α-linolenic acid, in addition to SFA, MUFA, and PUFA. Detrend was used for palmitoleic acid, while a combination of MSC and detrend was applied to palmitic acid. Stearic acid required a second-order derivation; only lauric acid needed no mathematical pretreatment to obtain the best results. The correlation coefficient for determining calibration (R^2^_C_) and prediction (R^2^_P_) was over 0.91, and RPD values were higher than 3.7 for lauric acid, myristic acid, oleic acid, linoleic acid, and α-linolenic acid and were observed as usable for the routine prediction of these fatty acids. A robust calibration for palmitic acid was achieved with an R^2^_C_ of 0.812, an R^2^_P_ of 0.877, and an RPD value of 2.66. The correlation coefficient for determining calibration (R^2^_C_ = 0.337) and prediction (R^2^_P_ = 0.345) was very low for palmitoleic acid, with an RPD value of 1.57. Stearic acid reached a low R^2^_C_ (0.579), R^2^_P_ (0.510), and RPD value (1.96) too. Consequently, the PLS models are not capable of predicting palmitoleic and stearic acid.

**Figure 5:**
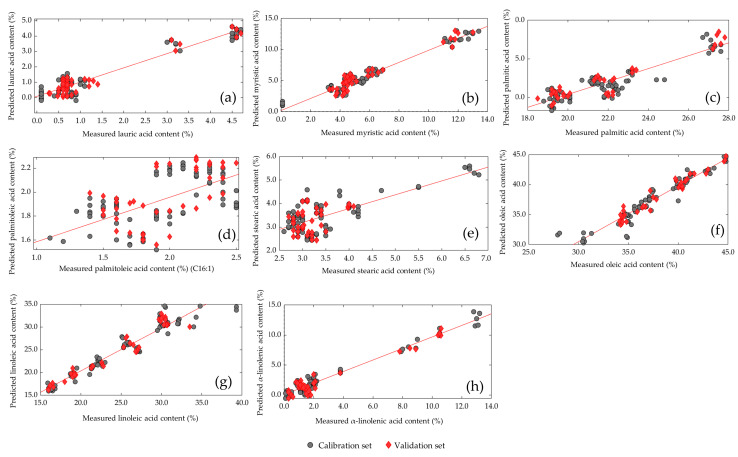
Comparison of the measured and predicted values of (**a**) lauric acid, (**b**) myristic acid, (**c**) palmitic acid, (**d**) palmitoleic acid, (**e**) stearic acid, (**f**) oleic acid, (**g**) linoleic acid, and (**h**) α-linoleic acid of living mealworm larvae in the calibration (•) and validation sets (♦).

Figure 5 shows the comparison between the measured and predicted values of lauric acid, myristic acid, palmitic acid, palmitoleic acid, stearic acid, oleic acid, linoleic acid, and α-linolenic acid in the calibration and validation sets for the chosen PLS models. The prediction models for fatty acids with the individual values of the different feeding groups can be viewed in the Supplementary Material (Figure S2). The statistical analysis shows that the fatty acid content of *Tenebrio molitor* larvae can be predicted. The results of the validation (Table 7) also showed that the NIR calibration equations were not similarly dependent on correlations between the total fat content and relative FA concentrations (R^2^_F_), as the correlation coefficient ranged between 0.019 and 0.568 and was, for instance, higher for oleic acid (R^2^_F_ = 0.527) than for stearic acid (R^2^_F_ = 0.071).

**Figure 6:**
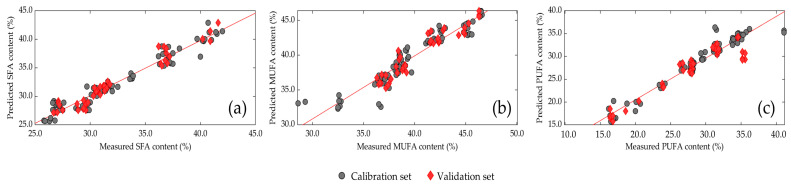
Comparison of the measured and predicted values of (**a**) saturated fatty acids (SFA), (**b**) monounsaturated fatty acids (MUFA), and (**c**) polyunsaturated fatty acids (PUFA) of living mealworm larvae in the calibration (•) and validation sets (♦).

The measurements and predictions for the chosen PLS models (Figure 6) were compared between SFA, MUFA, and PUFA. Based on the criteria described previously, the best model was selected due to a high RPD and R^2^_P_, a low RMSEP, and a limited number of latent variables (<10). Detailed prediction models with individual values for SFA, MUFA, and PUFA of each feeding group are available in the Supplementary Materials (Figure S3). The analysis of statistical data indicated that the SFA, MUFA, and PUFA content can be predicted in living *Tenebrio molitor* larvae (Table 6), but NIR models are more accurate when the raw spectra are mathematically preprocessed. The correlation coefficients were 0.948 (R^2^_C_) and 0.942 (R^2^_P_) for SFA, 0.886 (R^2^_C_) and 0.903 (R^2^_P_) for MUFA, and 0.943 (R^2^_C_) and 0878 (R^2^_P_) for PUFA. The RPD values (3.03–4.26) observed are usable for routine analysis.

## 4. Discussion

Insects represent a sustainable alternative protein source but also provide an appreciable amount of fat, which has usually a high PUFA content [8]. Consequently, the research on the nutritional requirements, metabolism, and transport of nutrients, and especially the utilization and storage of dietary lipids in insects, has become a very important topic [10]. Insects have tremendous plasticity in lipid accumulation and fatty acid profile which is mainly dependent on diet, developmental and larval stage, species, and environmental conditions (e.g., temperature, humidity, etc.) [34,35,36]. In this study, the fat content and FA composition of *Tenebrio molitor* larvae were analyzed to determine a suitable feeding substrate and to modify the FA content for achieving a healthier larva with a valuable FA composition. FAs perform several important functions in insects by acting as precursors for the synthesis of pheromones, an energy reserve, and an important component for the performance of metamorphosis [37,38]. The main FAs detected in mealworm larvae were, in particular, oleic, linoleic, and palmitic acid and are similar to those reported in previous studies [9,39]. Normally, commercially produced insects consist of a high amount of oleic and linoleic acid due to feeding with grains and grain by-products that are generally high in oleic and linoleic acid [40,41]. This is consistent with the results of this publication, as feeding with wheat bran led to high levels of these FAs. Diet can also change the FA composition of mealworm larvae, especially the content of α-linolenic and oleic acid. However, the FA composition of *Tenebrio molitor* larvae did not always reflect the diet, indicating that some physiological regulation of lipids in insects may appear [42]. Several insects show an extension pathway similar to that described for vertebrates, but unlike vertebrates, the larvae of *Tenebrio molitor* can synthesize linoleic and α-linolenic acid de novo, too [43,44]. Insects can also biosynthesize long-chain polyunsaturated FAs, such as eicosapentaenoic acid (EPA, C20:5) and docosahexaenoic acid (DHA, C22:6), from the C18 polyunsaturated FAs in their diet [43,45,46,47]. These two primary n-3 long-chain PUFA were not found in the larvae of *T. molitor* examined in this study. According to van Broekhoven et al., (2015) [24] and Siemianowska et al., (2013) [48], mealworm larvae that were raised on a substrate with a high protein and low carbohydrate content contained EPA, which was most likely acquired from their diet. However, since an enrichment of EPA and DHA could be achieved in the black soldier fly (*Hermetia illucens*) by the feeding of fish waste [49], it may also be feasible to accumulate EPA and DHA in *Tenebrio molitor* by feeding them an appropriate diet. A majority of insects acquire fatty acids through the absorption of dietary lipids in their midgut epithelium or from sugars produced by their enterocytes [50]. A higher inclusion level of flaxseed flour resulted in an increase in the concentrations of stearic and oleic acids, as well as a numerically high increase in the concentration of α-linolenic acid in mealworm larvae. Where the flaxseed flour inclusion amount is higher, these 18-carbon fatty acids might be more abundant as a result of microbial biohydrogenation, as occurs in sheep and cattle [51]. It has also been suggested that the consumption of carrots could influence the FA composition and ω6/ω3 ratio [24]. In the present work, carrots were added to all diets, so it was not possible to determine if carrot consumption would affect the larval composition. The shortest FA analyzed in this paper is lauric acid (C12:0) (see Table 3). However, the shorter FA capric acid (C10:0) was detected by Cito et al. (2017) [39] when mealworm larvae were raised on layer feed; the SFA content ranged between 26.3 and 41.4%. Similar SFA values were reported by Lawal et al. (2021) [52] when the diet was supplemented with flaxseed flour (26.9–28.4%), and Cito et al. (2017) [39] were able to achieve an SFA value of 28.8% when larvae were fed with wheat flour, oat flour, and yeast. Furthermore, a comparable SFA composition (29.6%) [39] was found in the larvae as in the present research from feeding pure wheat bran (29.3%). However, lower SFA values in *Tenebrio molitor* larvae fed only with wheat bran were also described by Ravzanaadii et al. (2012) [42] (22.3%). Studies by Giannetto et al. (2020) [53] and Meneguz et al. (2018) [54] have shown that a rearing substrate rich in polysaturated FAs can affect the FA profile, meaning that the insect larvae can become rich in saturated FAs. The fat content of mealworm larvae ranged between 5.7 and 16.1% and was similar to those reported in the literature [40,55,56]. The differences in the fat content of insects are normally due to the diet, rearing conditions, extraction method, and type of species [57]. All groups attained a higher lipid content compared to the initial larvae at an age of 8 weeks, regardless of the substrate. This is due to the progressive development having an influence on the nutritional composition, especially the fat content, of *T. molitor* larvae [58]. All diets in this study had a high survival rate (≥99%), making them suitable for *Tenebrio molitor* development. Our research clearly demonstrates that the fat content and fatty acid composition are hugely affected by diet, which was also observed in similar investigations [8,9,52,59]. Consequently, the differences in the fat content of *T. molitor* larvae observed in this research can be linked to the nutrient content of the larval diets on which they were reared. Van Broekhoven et al. (2015) showed that the fat content of *Tenebrio molitor* larvae was greatly influenced by the various starch and protein contents of their feeding substrates, suggesting that mealworm larvae fed with a low nutritional quality substrate possibly use their fat reserves for energy, thereby reducing their fat content [23]. 

In insects raised on diets with a high protein content or a variable protein/starch ratio, differences in the larval growth rate were noted. The growth rates and weight gain of *T. molitor* larvae were different based on diet treatments, depending on their contrasting differences in nutritional composition. Some previous studies demonstrated that the nutrient content and composition differ between and within insect species as a result of different diets and feeding patterns [8,52,59]; generally, low protein levels reduce growth rates [60,61]. The growth of insects is affected by the ratio of nutrients absorbed, and insects may use behavioral and post-ingestive mechanisms to compensate for nutrient deficiency [62,63]. A similar ratio between proteins and carbohydrates appears to be the best diet for beetles, but they may have eaten low-protein substrates at higher rates to ensure that they consumed sufficient nutrients [64]. However, a disproportional amount of protein content can also have a detrimental effect on larval growth. The protein content of the diets HPF8 (40.0%) and PPF6 (40.0%) is very high, but the larvae showed a reduced growth rate and weight gain. This may be due to the low carbohydrate contents of 18.4% (HPF8) and 29.5% (PPF6). According to other studies, the optimal macronutrient content varied depending on different factors (larval age, instar, diet, etc.) and should be between 61.3 and 79.2% for carbohydrates, between 14.4 and 25.4% for protein, and around 4.3 to 13.4% for fat [65,66,67]. As stated by Alves et al. (2016) [68], *Tenebrio molitor* larvae reared on diets (*acrocomia aculeata* pulp flour) with a high protein and low-fat content also exhibited the highest protein and fat levels. On the other hand, the fat content of mealworm larvae did not appear to be strongly influenced by the fat amount of the diet, as reported by Silva et al. (2021) [58]. Harsányi et al. (2020) [69] detected a minor variation in the protein and fat content of mealworm larvae, despite existing differences in dietary composition. The protein content of the diet should not influence the larval protein content when they are reared on a nutritionally balanced diet, as excessive protein is catabolized, which could be observed by the increase in the uric acid in the larval excreta [24]. As a result, mealworm larvae with a higher protein content may accumulate less fat due to diets with lower energy sources. This effect was also observed in locusts (*Locusta migratoria*) [70], which could explain the differences reported in other studies. However, there is no information on whether the ingestion rates differed between treatments. Furthermore, the diets used in this research were not isocaloric, so the larvae could have responded to changes in energy intake and different elements such as nitrogen contained in the substrate, as appears in several arthropods [71,72]. Consequently, the quality (including the total nitrogen content) of the larvae diet could influence the growth rate and body mass of *T. molitor* larvae. The last instars normally have the highest lipid reserves [73] and increase as larval development progresses [65] but decrease significantly in the adult since fat is needed as an energy source during pupation [74]. This might be the reason why the fat content of the larvae at the beginning of the experiment is much lower (5.7 ± 1.1 g/100 g) than the fat content of the larvae at the end (7.6–16.1 g/100 g) of the feeding trial. Additionally, other factors can also have a significant effect on the larvae’s nutritional composition. A number of environmental factors (temperature, humidity, light, etc.) have been shown to affect the development, growth, and nutritional composition of insects [22,34,36]. 

This paper is the first study to predict the fat content in living *Tenebrio molitor* larvae using near-infrared reflectance spectroscopy, with prediction models (R^2^_C_ = 0.975, R^2^_P_ = 0.986, RPD = 8.33) that are comparable with the analysis of fat content in pork (R^2^_C_ = 0.84–0.99, R^2^_P_ = 0.84–0.98) [19] or cheese (R^2^_C_ = 0.972–0.995, R^2^_P_ = 0.914–0.933) [75], and better than the prediction of fat content in rice (R^2^_C_ = 0.73–0.89, R^2^_P_ = 0.62–0.87) [20], beef (R^2^_C_ = 0.879, R^2^_P_ = 0.901, RPD = 2.84) [76], and nuts (R^2^_C_ = 0.96, R^2^ = 0.97, RPD = 5.61) [77]. As a complex matrix, measuring living mealworm larvae using NIRS is challenging, since the nutrient content of homogenized samples, such as insect flour [78], can be predicted more accurately than in intact samples [79]. A significant number of spectra peaks were detected at 1454 nm (combination of C-H stretching and first overtone stretching) and at 1930 nm (combination of N-H stretching and O-H stretching), mostly associated with protein, water, sugar, and starch concentrations [80,81]. The second overtone of C-H stretching at 1205 nm, and the first overtone of C-H stretching between 1727 and 1797 nm are usually associated with fat and fatty acid content [82,83]. The predictability of fat content in living mealworm larvae is very good, but it could probably be improved by creating a larger variety of data for fat content within the calibration, as other studies have shown if more data points are available, a higher model performance is reached [84,85]. Since the fat content of living *Tenebrio molitor* larvae can be predicted highly accurately, it may be possible to monitor the amount of fat in real-time online for the large-scale production of mealworm larvae, resulting in a significant advantage in terms of influencing the nutritional content of the larvae and achieving the desired nutrient profile at harvest time through specific feeding. Published studies also demonstrate the applicability of NIRS for FA profiling in different food and feed such as oilseeds [84], chicken breast [86], pork sausages [87], and fish oil [88]. The purpose of this study was to compare the performances of the calibration methods in order to investigate the possibility of using NIRS for measuring the FA composition of living mealworm larvae. This study is the first that demonstrated a very high degree of accuracy in predicting the fatty acid content of living mealworm larvae. The prediction results of validation demonstrated that the equation for oleic acid had the highest predictive ability (R^2^_P_ = 0.949, RPD = 5.55), followed by α-linolenic acid (R^2^_P_ = 0.945, RPD = 4.90), myristic acid (R^2^_P_ = 0.930, RPD = 4.11), linoleic acid (R^2^_P_ = 0.931, RPD = 3.98), and lauric acid (R^2^_P_ = 0.917, RPD = 3.73), and are valid for quality analysis. The palmitic acid model (R^2^_P_ = 0.877, RPD = 2.66) was usable for several research applications such as sample screening. A high predictive capability was performed for analyzing the SFA (R^2^_P_ = 0.942, RPD = 4.26), MUFA (R^2^_P_ = 0.903, RPD = 4.00), and PUFA (R^2^_P_ = 0.878, RPD = 3.03) content in *Tenebrio molitor* larvae. However, no suitable predictive model for palmitoleic (R^2^_P_ = 0.345, RPD = 1.57) and stearic (R^2^_P_ = 0.509, RPD = 1.96) acids could be established. A similar model with low prediction accuracy for stearic acid (R^2^_P_ = 0.49, RPD = 1.4) in soybeans was developed by Kovalenko et al. (2006) [84]. However, the author was also able to establish good prediction models for palmitic acid (R^2^_P_ = 0.80, RPD = 2.2) and SFA (R^2^_P_ = 0.91, RPD = 3.3) in soybeans [84]. There is a large variance in the prediction ability of NIR models attributed to the standard deviation of reference data obtained from calibration sets. As a result, introducing samples with extremely high and low palmitoleic and stearic acid values into calibration sets may increase the accuracy of the prediction [84]. The coefficient for determining the correlation of relative FA concentration with the fat content (R^2^_F_) of mealworm larvae had a wide range (0.019–0.568), which suggests that not all fatty acids have the same dependence on the total fat content. Prediction values (R^2^_P_) of the NIR calibration models for α-linolenic acid were high (0.945), whereas the correlation coefficients between the fat content and α-linolenic acid in *Tenebrio molitor* larvae were very low (R^2^_F_ = 0.019). This indicates that the calibration methods of NIRS used in this research might have gained information from individual FA absorption bands rather than from broader total fat absorption bands. This has also been observed in a study by Kovalenko et al. (2006) [84].

## 5. Conclusions

Diet has a significant effect on the nutritional composition of *Tenebrio molitor* larvae. It is important to emphasize the significance of diet when influencing the fat content and fatty acid profile of mealworm larvae, although physiological processes could dampen fatty acid enrichment. However, high levels of fatty acids in the substrate cause the accumulation of a higher content of fatty acids in the larval body. However, a high fat content in the diet does not significantly increase the fat content of the larvae since other factors and nutrients (e.g., carbohydrates and proteins) also have a major influence on the larval composition. Furthermore, this study proves that near-infrared reflectance spectroscopy can be used to predict the total fat and fatty acid content of living *Tenebrio molitor* larvae and could therefore be an alternative to conventional analytical methods; no larvae have to be killed for measurement since samples can be analyzed in their original form.

## Figures and Tables

**Table 1:** Compositional amount (%) of experimental diets for *Tenebrio molitor* larvae.

Group	Substrate Amount (%)						
	Coconut Flour (CF)	Flaxseed Flour (FSF)	Rose Hip Hulls (RHH)	Grape Pomace (GP)	Hemp Protein Flour (HPF)	Pea Protein Flour (PPF)	Wheat Bran (WB)
CF5	3.4	-	-	-	-	-	96.6
CF10	23.8	-	-	-	-	-	76.2
CF15	46.2	-	-	-	-	-	53.8
CF20	68.6	-	-	-	-	-	31.4
FSF5	-	1.1	-	-	-	-	98.9
FSF10	-	18.7	-	-	-	-	81.3
FSF15	-	36.4	-	-	-	-	63.6
FSF20	-	54.1	-	-	-	-	45.9
GP4	-	-	-	42.5	-	-	57.5
HPF5	-	-	-	-	14.5	-	85.5
HPF8	-	-	-	-	71.5	-	28.5
RHH4	-	-	36.4	-	-	-	63.6
PPF5	-	-	-	-	-	7.8	92.2
PPF6	-	-	-	-	-	38.6	61.4
WB (Control)	-	-	-	-	-	-	100.0

CF5: coconut flour and wheat bran (5% fat); CF10: coconut flour and wheat bran (10% fat); CF15: coconut flour and wheat bran (15% fat); CF20: coconut flour and wheat bran (20% fat); FSF5: flaxseed flour and wheat bran (5% fat); FSF10: flaxseed flour and wheat bran (10% fat); FSF15: flaxseed flour and wheat bran (15% fat); FSF20: flaxseed flour and wheat bran (20% fat); GP4: grape pomace and wheat bran (5% fat); HPF5: hemp protein flour and wheat bran (5% fat); HPF8: hemp protein flour and wheat bran (8% fat); RHH4: rose hip hulls and wheat bran (4% fat); PPF5: pea protein flour and wheat bran (5% fat); PPF6: pea protein flour and wheat bran (6% fat); and WB: wheat bran (control).

**Table 2:** Nutritional composition of the substrates on a fresh weight (FW) basis (%) used for *Tenebrio molitor* diets.

Substrate	Moisture (%)	Protein (% FW)	Fat (% FW)	Carbohydrate (% FW)	Fiber (% FW)	Ash (% FW)
CF5	12.0	14.9	5.0	44.9	17.6	5.6
CF10	11.2	15.2	10.0	43.3	15.6	4.8
CF15	10.4	15.4	15.0	41.8	13.5	3.9
CF20	9.6	15.7	20.0	40.2	11.5	3.0
FSF5	12.0	15.0	5.0	44.8	17.6	5.7
FSF10	11.2	16.4	10.0	41.3	15.6	5.5
FSF15	10.5	17.8	15.0	37.7	13.7	5.3
FSF20	9.8	19.2	20.0	34.2	11.7	5.1
GP4	11.0	12.0	4.4	51.0	16.7	5.0
HPF5	11.6	20.0	5.3	39.6	18.0	5.5
HPF8	9.9	40.0	7.8	18.4	19.3	4.6
RHH4	12.0	10.8	3.5	51.0	17.0	5.7
PPF5	11.3	20.0	5.0	41.9	16.6	5.3
PPF6	8.4	40.0	6.0	29.5	12.5	3.6
WB (Control)	12.0	14.9	4.7	45.0	17.7	5.7

CF5: coconut flour and wheat bran (5% fat); CF10: coconut flour and wheat bran (10% fat); CF15: coconut flour and wheat bran (15% fat); CF20: coconut flour and wheat bran (20% fat); FSF5: flaxseed flour and wheat bran (5% fat); FSF10: flaxseed flour and wheat bran (10% fat); FSF15: flaxseed flour and wheat bran (15% fat); FSF20: flaxseed flour and wheat bran (20% fat); GP4: grape pomace and wheat bran (5% fat); HPF5: hemp protein flour and wheat bran (5% fat); HPF8: hemp protein flour and wheat bran (8% fat); RHH4: rose hip hulls and wheat bran (4% fat); PPF5: pea protein flour and wheat bran (5% fat); PPF6: pea protein flour and wheat bran (6% fat); and WB: wheat bran (control).

**Table 3:** Fatty acid composition of *Tenebrio molitor* larvae on a dry matter (DM) basis (relative% of total fatty acids). Data are presented as the mean ± standard deviation, *n* = 2.

Fatty Acid	Group															
	Start	CF5	CF10	CF15	CF20	FSF5	FSF10	FSF15	FSF20	GP4	HPF5	HPF8	RHH4	PPF5	PPF6	WB
Lauric acid (C12:0)	n.d.	0.7 ± 0.1	4.6 ± 0.1	4.7 ± 0.1	3.2 ± 0.1	0.6 ± 0.0	0.6 ± 0.0	n.d.	0.5 ± 0.1	0.7 ± 0.1	0.7 ± 0.1	0.9 ± 0.1	0.7 ± 0.0	1.0 ± 0.1	n.d.	0.8 ± 0.1
Myristic acid (C14:0)	3.3 ± 0.0	4.8 ± 0.1	12.2 ± 0.7	12.9 ± 0.7	11.4 ± 0.4	3.3 ± 0.1	4.4 ± 0.1	n.d.	4.2 ± 0.1	6.4 ± 0.6	5.0 ± 0.6	4.4 ± 0.1	6.1 ± 0.5	4.4 ± 0.1	5.6 ± 0.4	3.8 ± 0.2
Palmitic acid (C16:0)	23.0 ± 2.6	22.2 ± 0.5	21.4 ± 0.2	20.1 ± 0.0	19.2 ± 0.1	19.3 ± 0.2	19.3 ± 0.7	22.2 ± 0.8	19.5 ± 0.5	27.1 ± 0.6	22.5 ± 0.7	19.2 ± 0.3	27.1 ± 0.5	23.1 ± 0.2	21.4 ± 0.2	21.7 ± 1.0
Palmitoleic acid (C16:1)	1.4 ± 0.5	1.6 ± 0.1	2.4 ± 0.1	1.5 ± 0.0	1.7 ± 0.1	2.1 ± 0.1	1.9 ± 0.0	1.4 ± 0.1	1.8 ± 0.1	2.3 ± 0.1	2.2 ± 0.1	2.5 ± 0.1	2.0 ± 0.1	2.3 ± 0.1	2.1 ± 0.1	1.6 ± 0.0
Stearic acid (C18:0)	4.7 ± 1.2	2.9 ± 0.0	3.1 ± 0.1	2.7 ± 0.1	3.2 ± 0.1	3.5 ± 0.0	3.4 ± 0.0	4.2 ± 0.0	4.0 ± 0.0	2.9 ± 0.1	3.4 ± 0.1	2.9 ± 0.1	2.8 ± 0.1	3.0 ± 0.0	6.7 ± 0.2	3.1 ± 0.1
Oleic acid (C18:1 ω9)	31.3 ± 4.9	39.8 ± 0.4	36.1 ± 0.5	41.2 ± 0.4	44.8 ± 0.1	43.1 ± 0.7	40.8 ± 0.2	37.1 ± 0.0	40.3 ± 0.7	36.8 ± 0.7	34.7 ± 0.6	35.7 ± 0.3	34.4 ± 0.7	35.0 ± 0.4	30.5 ± 0.2	37.4 ± 0.6
Linoleic acid (C18:2 ω6)	34.8 ± 6.3	27.2 ± 0.3	19.1 ± 0.4	16.5 ± 0.6	16.3 ± 0.6	25.9 ± 0.2	21.3 ± 0.3	22.1 ± 0.5	19.2 ± 0.4	22.7 ± 0.6	29.5 ± 0.6	30.8 ± 0.1	25.3 ± 0.3	29.8 ± 0.1	32.1 ± 0.1	30.3 ± 0.4
α-Linolenic acid (C18:3 ω3)	1.4 ± 0.6	0.9 ± 0.1	1.0 ± 0.1	0.5 ± 0.1	0.2 ± 0.1	2.1 ± 0.1	8.4 ± 0.7	13.0 ± 0.3	10.5 ± 0.0	1.1 ± 0.0	2.0 ± 0.0	3.8 ± 0.0	1.4 ± 0.1	1.4 ± 0.0	1.7 ± 0.1	1.3 ± 0.1
∑ SFA	31.0 ± 1.4	30.6 ± 0.7	41.4 ± 0.9	40.4 ± 0.9	40.0 ± 0.4	26.8 ± 0.2	27.6 ± 0.8	26.3 ± 0.7	28.2 ± 0.4	36.2 ± 1.4	31.6 ± 1.3	27.3 ± 0.3	36.9 ± 0.9	31.6 ± 1.3	33.6 ± 0.4	29.3 ± 1.1
∑ MUFA	32.8 ± 5.4	41.4 ± 0.5	38.6 ± 0.6	42.7 ± 0.4	46.5 ± 0.1	45.2 ± 0.5	42.7 ± 0.2	38.5 ± 0.1	42.1 ± 0.8	39.1 ± 0.8	36.4 ± 0.7	38.2 ± 0.4	36.4 ± 0.5	36.9 ± 0.7	32.6 ± 0.1	39.1 ± 0.6
∑ PUFA	36.2 ± 6.8	28.0 ± 0.1	20.1 ± 0.3	16.9 ± 0.5	16.6 ± 0.5	28.0 ± 0.3	29.7 ± 1.0	35.1 ± 0.8	29.7 ± 0.4	23.8 ± 0.6	31.5 ± 0.6	34.5 ± 0.1	26.7 ± 0.3	31.5 ± 0.6	33.8 ± 0.3	31.7 ± 0.5

n.d.: not detected; Start: fatty acid content of the larvae at the beginning of the experiment; SFA: saturated fatty acids; MUFA: monounsaturated fatty acids; PUFA: polyunsaturated fatty acids; CF5: coconut flour and wheat bran (5% fat); CF10: coconut flour and wheat bran (10% fat); CF15: coconut flour and wheat bran (15% fat); CF20: coconut flour and wheat bran (20% fat); FSF5: flaxseed flour and wheat bran (5% fat); FSF10: flaxseed flour and wheat bran (10% fat); FSF15: flaxseed flour and wheat bran (15% fat); FSF20: flaxseed flour and wheat bran (20% fat); GP4: grape pomace and wheat bran (5% fat); HPF5: hemp protein flour and wheat bran (5% fat); HPF8: hemp protein flour and wheat bran (8% fat); RHH4: rose hip hulls and wheat bran (4% fat); PPF5: pea protein flour and wheat bran (5% fat); PPF6: pea protein flour and wheat bran (6% fat); and WB: wheat bran (control).

**Table 4:** Fat content on a fresh weight (FW) basis (g/100 g) of the living *Tenebrio molitor* larvae in the calibration (*n* = 80) and validation (*n* = 40) sets.

Statistics	Calibration Set	Validation Set
	Fat (g/100 g of FW)	Fat (g/100 g of FW)
Mean	12.0	12.1
Minimum	7.4	7.3
Maximum	16.2	16.2
SD	2.3	2.3

SD: standard deviation.

**Table 5:** Statistics of the PLS model prediction of the fat content in living *Tenebrio molitor* larvae.

Item	Mathematical Treatment	No. of Latent Variables	Calibration Set		Validation Set		
			R^2^_C_	RMSEC	R^2^_P_	RMSEP	RPD
Fat	None	6	0.955	0.482	0.967	0.431	5.34
	MSC	5	0.962	0.443	0.969	0.412	5.58
	MC	6	0.975	0.355	0.986	0.276	8.33
	1D	4	0.949	0.512	0.954	0.502	4.58
	2D	5	0.958	0.461	0.961	0.467	4.93

R^2^_C_: coefficient of determination for calibration; R^2^_P_: coefficient of determination for prediction; RMSEC: root mean square error of calibration; RMSEP: root mean square error of prediction; RPD: ratio of performance deviation; MSC: multiple scatter correction; MC: mean centering; and 1D and 2D: first and second derivative.

**Table 6:** Fatty acid content on a dry weight (DW) basis (%) of living *Tenebrio molitor* larvae for the calibration (*n* = 80) and validation sets (*n* = 40).

Fatty Acid (% of DW)	Calibration Set				Validation Set			
	Minimum	Maximum	Mean	SD	Minimum	Maximum	Mean	SD
Lauric acid (C12:0)	0.4	4.7	1.2	1.5	0.3	4.9	1.1	1.4
Myristic acid (C14:0)	3.2	13.4	5.8	3.4	3.3	13.0	5.6	3.3
Palmitic acid (C16:0)	18.8	27.6	21.8	2.5	18.5	27.8	22.0	2.7
Palmitoleic acid (C16:1)	1.1	2.5	1.9	0.4	1.3	2.4	1.8	0.4
Stearic acid (C18:0)	2.6	6.8	3.5	1.0	2.9	6.5	3.6	1.0
Oleic acid (C18:1 ω9)	27.9	44.8	37.5	4.0	28.7	45.2	37.3	4.2
Linoleic acid (C18:2 ω6)	15.9	39.3	25.3	5.9	16.0	35.4	25.1	5.6
α-Linolenic acid (C18:3 ω3)	0.1	13.2	3.2	3.8	0.3	13.5	3.3	4.0
SFA	25.8	42.0	32.4	4.8	26.2	41.6	32.2	4.6
MUFA	28.6	46.6	39.5	4.1	30.5	46.3	39.4	4.2
PUFA	16.2	41.1	28.2	6.2	16.4	40.2	28.5	6.4

SD: standard deviation; SFA: saturated fatty acids; MUFA: monounsaturated fatty acids; and PUFA: polyunsaturated fatty acids.

**Table 7:** Statistics of NIR prediction models for fatty acid content in living mealworm larvae.

Fatty Acid	Mathematical Treatment	No. of Latent Variables	Calibration Set		Validation Set			
			R^2^_C_	RMSEC	R^2^_P_	RMSEP	RPD	R^2^_F_
Lauric acid (C12:0)	None	8	0.915	0.418	0.917	0.375	3.73	0.253
Myristic acid (C14:0)	MSC	8	0.947	0.773	0.930	0.803	4.11	0.331
Palmitic acid (C16:0)	MSC + Detrend	8	0.812	1.077	0.877	1.012	2.66	0.269
Palmitoleic acid (C16:1)	Detrend	8	0.337	0.290	0.345	0.255	1.57	0.156
Stearic acid (C18:0)	2D	8	0.579	0.638	0.510	0.509	1.96	0.071
Oleic acid (C18:1 ω9)	MSC	8	0.922	1.104	0.949	0.756	5.55	0.527
Linoleic acid (C18:2 ω6)	MSC	8	0.925	1.602	0.931	1.411	3.98	0.568
α-Linolenic acid (C18:3 ω3)	MSC	8	0.964	0.713	0.945	0.817	4.90	0.019
SFA	MSC	8	0.948	1.088	0.942	1.081	4.26	0.091
MUFA	MSC	8	0.886	1.371	0.903	1.050	4.00	0.511
PUFA	MSC	8	0.943	1.466	0.878	2.115	3.03	0.282

SFA: saturated fatty acids; MUFA: monounsaturated fatty acids; PUFA: polyunsaturated fatty acids; R^2^_C_: coefficient of determination for calibration; R^2^_P_: coefficient of determination for prediction; R^2^_F_: coefficient of determination describing the correlation of relative FA concentration with the total fat content; RMSEC: root mean square error of calibration; RMSEP: root mean square error of prediction; RPD: ratio of performance deviation; MSC: multiple scatter correction; Detrend; and 2D: second derivative.

## Data Availability

The data presented in this study are available on request from the corresponding author.

## Supplementary Materials

The following supporting information can be downloaded at: https://www.mdpi.com/article/10.3390/insects14020114/s1, Figure S1: Average NIR raw spectra of living *Tenebrio molitor* larvae from all samples (*n* = 5) of all groups (*n* = 15); Figure S2: Comparison of measured and predicted values of (a) lauric acid, (b) myristic acid, (c) palmitic acid, (d) palmitoleic acid, (e) stearic acid, (f) oleic acid, (g) linoleic acid and (h) α-linoleic acid of living mealworm larvae of each feeding group; Figure S3: Comparison of measured and predicted values of (a) saturated fatty acids (SFA), (b) monounsaturated fatty acids (MUFA) and (c) polyunsaturated fatty acids (PUFA) of living mealworm larvae of each feeding group; Table S1: Nutritional composition (as specified by the manufacturer) of substrates on a fresh weight (FW) basis (%) used for *Tenebrio molitor* diets; Table S2: Fatty acid composition of the substrates on a dry matter (DM) basis (%) used for *Tenebrio molitor* diets; Table S3: Calculated fatty acid composition on a dry matter (DM) basis (%) of the different groups used for *Tenebrio molitor* feeding experiment.
