# Supplementary material for: Near-Infrared Reflectance Spectroscopy for Quantitative Analysis of Fat and Fatty Acid Content in Living Tenebrio molitor Larvae to Detect the Influence of Substrate on Larval Composition
Source: Insects. 2023 Jan 23;14(2):114. doi: 10.3390/insects14020114 (PMC9964368; doi:10.3390/insects14020114)
Supplement: Supplementary file 1 [file insects-14-00114-s001.zip › insects-2150105-supplementary.pdf]

insects

MDPI

Article

Near-Infrared Reflectance Spectroscopy for Quantitative Analysis of Fat and Fatty Acid Content in Living *Tenebrio molitor* Larvae to Detect the Influence of Substrate on Larval Composition

Nina Kröncke *, Monique Neumeister and Rainer Benning

Institute of Food Technology and Bioprocess Engineering, University of Applied Sciences Bremerhaven, 27568 Bremerhaven, Germany
* Correspondence: nkroencke@hs-bremerhaven.de; Tel.: +49-(0)4-714-823-269

Table S1. Nutritional composition (as specified by the manufacturer) of substrates on a fresh weight (FW) basis () used for *Tenebrio molitor* diets.

Copyright: © 2023 by the authors. Licensee MDPI, Basel, Switzerland. This article is an open access article distributed under the terms and conditions of the Creative Commons Attribution (CC BY) license (https://creativecommons.org/licenses/by/4.0/).

Substrate	Moisture (%)	Protein (% of FW)	Fat (% of FW)	Carbohydrate (% of FW)	Fiber (% of FW)	Ash (% of FW)	Manufacturer
Coconut flour	8.5	16.0	27.0	38.0	8.7	1.8	Fischmix, Iserlohn, Germany
Flaxseed flour	7.9	22.9	33.0	25.0	6.6	4.6	Fischmix, Iserlohn, Germany
Grape pomace	9.6	8.0	3.9	59.1	15.4	4.0	Holger Senger Vertrieb von Naturrohstoffen e. K., Dransfeld, Germany
Rose hip hulls	12.0	3.5	1.5	61.5	15.8	5.7	Holger Senger Vertrieb von Naturrohstoffen e. K., Dransfeld, Germany
Hemp protein flour	9.0	50.0	9.0	7.8	20.0	4.2	Demeterhof Schwab GmbH & Co. KG, Windsbach, Germany
Pea protein flour	2.7	80.0	8.0	4.9	4.2	0.2	Raab Vitalfood GmbH, Rohrbach, Germany
Wheat bran	12.0	14.9	4.7	45.0	17.7	5.7	Roland Mills United GmbH & Co. KG, Bremen, Germany

Table S2. Fatty acid composition of the substrates on a dry matter (DM) basis (relative % of total fatty acids) used for *Tenebrio molitor* diets. Data are presented as mean ± standard deviation, *n* = 2.

**Fatty acid**	**Substrate**						
	Coconut flour	Flaxseed flour	Grape pomace	Rose hip hulls	Hemp protein flour	Pea protein flour	Wheat bran
Lauric acid (C12:0)	50.8±0.2	0.2±0.0	0.4±0.0	3.0±0.1	n. d.	3.4±0.1	n. d.
Myristic acid (C14:0)	18.3±0.3	0.4±0.1	0.4±0.0	2.4±0.1	0.7±0.0	1.2±0.1	0.6±0.1
Palmitic acid (C16:0)	10.9±0.1	8.2±0.3	26.2±0.6	23.7±0.2	12.4±0.1	24.6±0.2	22.7±0.3
Palmitoleic acid (C16:1)	n. d.	0.3 ±0.1	1.6±0.1	1.3±0.0	n. d.	n. d.	1.2±0.0
Stearic acid (C18:0)	2.7±0.1	5.5±0.1	4.3±0.1	5.6±0.2	3.0±0.1	5.2±0.5	3.0±0.0
Oleic acid (C18:1 ω9)	14.9±0.2	24.8±0.6	16.8±0.2	24.1±0.0	13.7±0.1	35.5±0.2	20.5±0.4
Linoleic acid (C18:2 ω6)	2.4±0.1	17.1±0.7	46.8±0.5	30.0±0.0	56.7±0.6	26.0±0.2	50.0±0.2
α-Linolenic acid (C18:3 ω3)	n. d.	43.5±0.3	2.3±0.0	9.9±0.3	13.6±0.6	4.0±0.0	2.2±0.0
**∑ SFA**	**82.7±0.0**	**14.3±0.3**	**32.6±0.4**	**34.7±0.3**	**16.1±0.2**	**34.5±0.4**	**26.2±0.2**
**∑ MUFA**	**14.9±0.2**	**25.1±0.7**	**18.4±0.6**	**25.4±0.0**	**13.7±0.1**	**35.5±0.2**	**21.7±0.4**
**∑ PUFA**	**2.4±0.1**	**60.6±0.4**	**49.9±0.5**	**39.9±0.3**	**70.3±0.1**	**30.0±0.2**	**52.1±0.2**

n. d.: not detected; SFA: saturated fatty acids; MUFA: monounsaturated fatty acids; PUFA: polyunsaturated fatty acids.

Table S3. Calculated fatty acid composition on a dry matter (DM) basis (relative % ot total fatty acids) of the different groups used for *Tenebrio molitor* feeding experiment.

**Fatty acid (% DM)**	**Group**													
	CF5	CF10	CF15	CF20	FSF5	FSF10	FSF15	FSF20	GP4	HPF5	HPF8	RHH4	PPF5	PPF6
Lauric acid (C12:0)	0.7	12.1	23.5	34.9	0.0	0.1	0.1	0.1	0.2	n. d.	n. d.	1.2	0.3	1.3
Myristic acid (C14:0)	0.8	4.8	8.7	12.7	0.6	0.5	0.5	0.5	0.5	0.6	0.7	1.3	0.6	0.8
Palmitic acid (C16:0)	22.5	19.9	17.2	14.6	22.5	19.9	17.4	14.8	24.2	21.2	15.3	23.1	22.8	23.4
Palmitoleic acid (C16:1)	1.2	0.9	0.7	0.4	1.2	1.1	0.9	0.7	1.4	1.1	0.4	1.3	1.1	0.8
Stearic acid (C18:0)	3.0	2.9	2.9	2.8	3.0	3.5	3.9	4.3	3.6	3.0	3.0	4.1	3.2	3.9
Oleic acid (C18:1 ω9)	20.4	19.2	17.9	16.7	20.5	21.3	22.1	22.8	18.9	19.5	15.6	22.0	21.7	26.3
Linoleic acid (C18:2 ω6)	49.3	38.6	28.0	17.3	49.5	43.7	37.9	32.1	48.5	50.9	54.7	41.8	48.0	40.7
α-Linolenic acid (C18:3 ω3)	2.2	1.7	1.2	0.7	2.7	9.9	17.2	24.5	2.2	3.9	10.4	5.4	2.4	2.9
**∑ SFA**	**26.9**	**39.6**	**52.3**	**64.9**	**26.1**	**23.9**	**21.9**	**19.8**	**32.6**	**24.7**	**19.0**	**29.7**	**26.8**	**29.4**
**∑ MUFA**	**21.6**	**20.1**	**18.6**	**17.1**	**21.8**	**22.4**	**23.0**	**23.6**	**18.4**	**20.5**	**16.0**	**23.2**	**22.8**	**27.0**
**∑ PUFA**	**51.4**	**40.3**	**29.2**	**18.0**	**52.2**	**53.7**	**55.2**	**56.7**	**49.0**	**54.8**	**65.1**	**47.1**	**50.4**	**43.6**

n. d.: not detected; CF5: coconut flour and wheat bran (5% fat); CF10: coconut flour and wheat bran (10% fat); CF15: coconut flour and wheat bran (15% fat); CF20: coconut flour and wheat bran (20% fat); FSF5: flaxseed flour and wheat bran (5% fat); FSF10: flaxseed flour and wheat bran (10% fat); FSF15: flaxseed flour and wheat bran (15% fat); FSF20: flaxseed flour and wheat bran (20% fat); GP4: grape pomace and wheat bran (5% fat); HPF5: hemp protein flour and wheat bran (5% fat); HPF8: hemp protein flour and wheat bran (8% fat); RHH4: rose hip hulls and wheat bran (4% fat); PPF5: pea protein flour and wheat bran (5% fat); PPF6: pea protein flour and wheat bran (6% fat); WB: wheat bran (control); SFA: saturated fatty acids; MUFA: monounsaturated fatty acids; PUFA: polyunsaturated fatty acids.

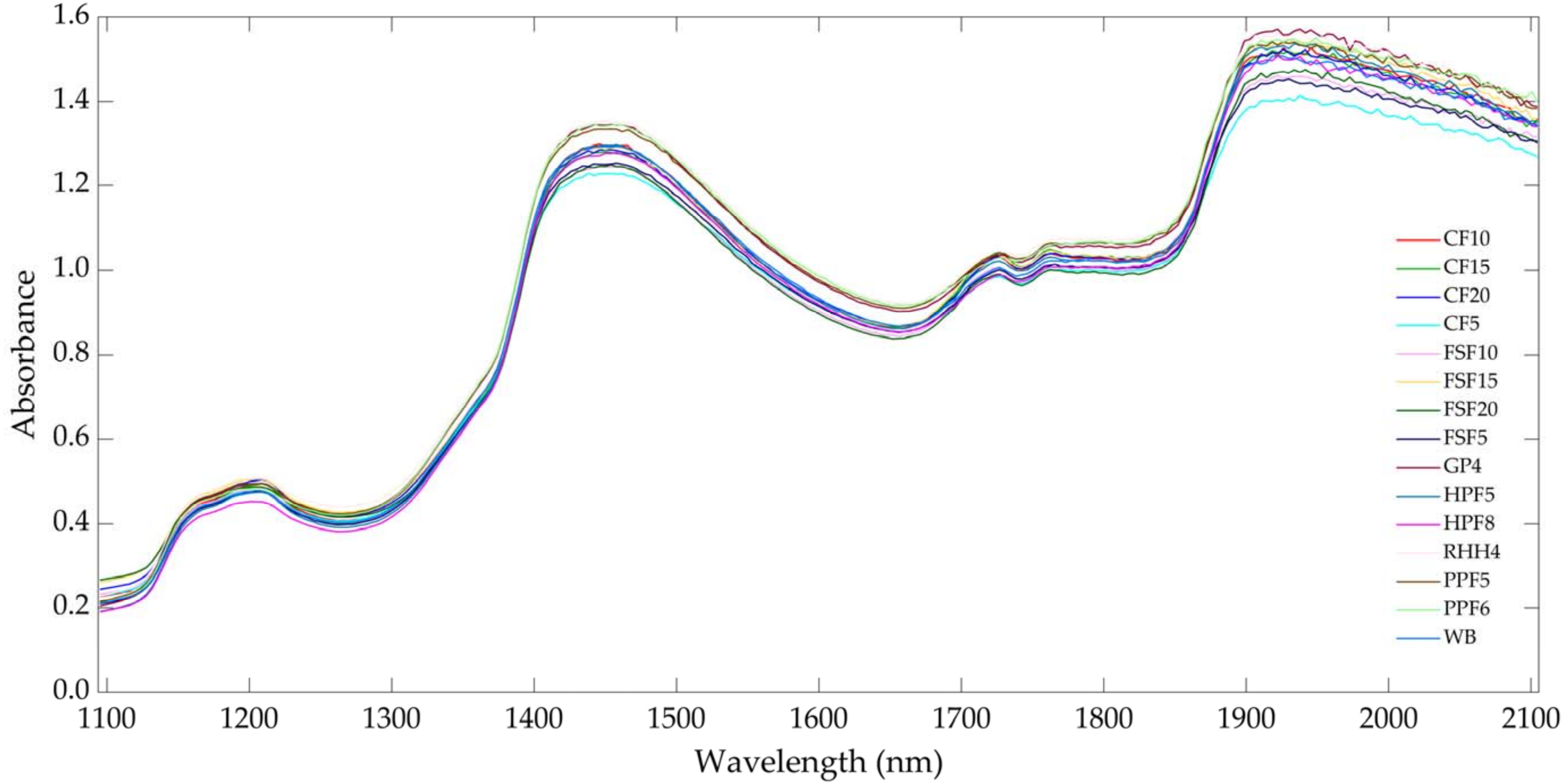

Figure S1. Average NIR raw spectra of living *Tenebrio molitor* larvae from all samples (*n* = 5) of all groups (*n* = 15), CF5: coconut flour and wheat bran (5% fat); CF10: coconut flour and wheat bran (10% fat); CF15: coconut flour and wheat bran (15% fat); CF20: coconut flour and wheat bran (20% fat); FSF5: flaxseed flour and wheat bran (5% fat); FSF10: flaxseed flour and wheat bran (10% fat); FSF15: flaxseed flour and wheat bran (15% fat); FSF20: flaxseed flour and wheat bran (20% fat); GP4: grape pomace and wheat bran (5% fat); HPF5: hemp protein flour and wheat bran (5% fat); HPF8: hemp protein flour and wheat bran (8% fat); RHH4: rose hip hulls and wheat bran (4% fat); PPF5: pea protein flour and wheat bran (5% fat); PPF6: pea protein flour and wheat bran (6% fat); WB: wheat bran (control).

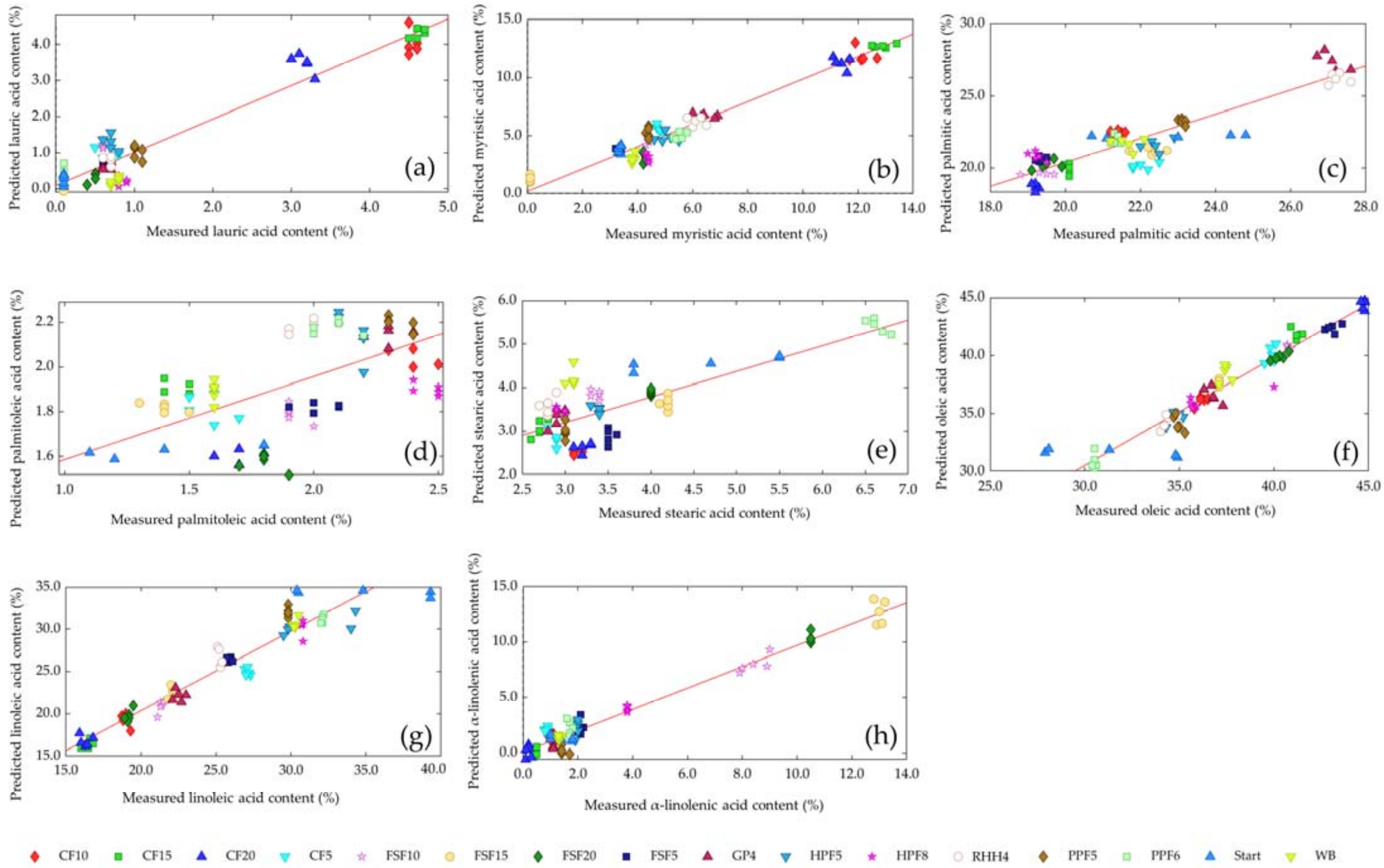

Figure S2. Comparison of measured and predicted values of (a) lauric acid, (b) myristic acid, (c) palmitic acid, (d) palmitoleic acid, (e) stearic acid, (f) oleic acid, (g) linoleic acid and (h) α-linoleic acid of living mealworm larvae of each feeding group: ▲ Start: larvae at the beginning of the experiment; ▼ CF5: coconut flour and wheat bran (5% fat); ♦ CF10: coconut flour and wheat bran (10% fat); ■ CF15: coconut flour and wheat bran (15% fat); ▲ CF20: coconut flour and wheat bran (20% fat); ■ FSF5: flaxseed flour and wheat bran (5% fat); ★ FSF10: flaxseed flour and wheat bran (10% fat); • FSF15: flaxseed flour and wheat bran (15% fat); ♦ FSF20: flaxseed flour and wheat bran (20% fat); ▲ GP4: grape pomace and wheat bran (5% fat); ▼ HPF5: hemp protein flour and wheat bran (5% fat); ★HPF8: hemp protein flour and wheat bran (8% fat); • RHH4: rose hip hulls and wheat bran (4% fat); ♦ PPF5: pea protein flour and wheat bran (5% fat); ■ PPF6: pea protein flour and wheat bran (6% fat); ▼ WB: wheat bran (control).

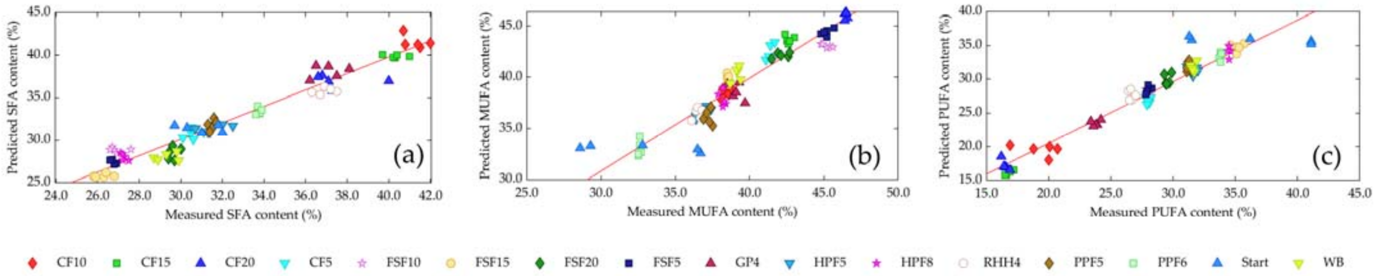

Figure S3. Comparison of measured and predicted values of (a) saturated fatty acids (SFA), (b) monounsaturated fatty acids (MUFA) and (c) polyunsaturated fatty acids (PUFA) of living mealworm larvae of each feeding group: ▲ Start: larvae at the beginning of the experiment; ▼ CF5: coconut flour and wheat bran (5% fat); ♦ CF10: coconut flour and wheat bran (10% fat); ■ CF15: coconut flour and wheat bran (15% fat); ▲ CF20: coconut flour and wheat bran (20% fat); ■ FSF5: flaxseed flour and wheat bran (5% fat); ★ FSF10: flaxseed flour and wheat bran (10% fat); • FSF15: flaxseed flour and wheat bran (15% fat); ♦ FSF20: flaxseed flour and wheat bran (20% fat); ▲ GP4: grape pomace and wheat bran (5% fat); ▼ HPF5: hemp protein flour and wheat bran (5% fat); ★HPF8: hemp protein flour and wheat bran (8% fat); • RHH4: rose hip hulls and wheat bran (4% fat); ♦ PPF5: pea protein flour and wheat bran (5% fat); ■ PPF6: pea protein flour and wheat bran (6% fat); ▼ WB: wheat bran (control).
